# Incomplete Kawasaki disease with multivessel giant coronary aneurysms and refractory thrombosis in an infant; a case report

**DOI:** 10.1093/ehjcr/ytag028

**Published:** 2026-01-22

**Authors:** Mohamed Abdelaal, Mustafa AlQbandi

**Affiliations:** Department of Pediatric Cardiology, Chest Diseases Hospital, Jamal Abdul Nasser Street, Shuwaikh Administrative Area, Shuwaikh, Al Asimah, Kuwait 70030, Kuwait; Department of Pediatric Cardiology, Chest Diseases Hospital, Jamal Abdul Nasser Street, Shuwaikh Administrative Area, Shuwaikh, Al Asimah, Kuwait 70030, Kuwait

**Keywords:** Kawasaki disease, Giant coronary aneurysm, Thrombosis, Antithrombotic therapy, Percutaneous coronary intervention, Direct oral anticoagulant, Case report

## Abstract

**Background:**

Kawasaki disease (KD) is an acute vasculitis of childhood with risk of coronary artery aneurysm (CAA) formation. Giant CAAs, thrombosis, and the need for revascularization in infancy are uncommon and clinically challenging.

**Case summary:**

A 9-month-old infant presented with persistent fever for 3 weeks before diagnosis and incomplete KD features. Echocardiography and computed tomographic coronary angiography revealed multivessel giant CAAs involving the left anterior descending (LAD), right coronary artery, and left circumflex (LCX) arteries, with multiple thrombi and a left atrial appendage thrombus. Alteplase was given in the intensive care unit for three cycles without thrombus resolution. A catheter-based attempt to recanalize the occluded LCX was unsuccessful. The patient was discharged on triple therapy with aspirin, clopidogrel, and rivaroxaban.

**Discussion:**

This case highlights the consequences of delayed recognition of incomplete KD, the limited efficacy of thrombolysis with alteplase in coronary thrombosis secondary to KD, the technical limitations and uncertain durability of percutaneous coronary intervention (PCI) in KD-related lesions during infancy, and evolving antithrombotic strategies, including the emerging role of direct oral anticoagulants for giant CAAs.

**Conclusion:**

Earlier diagnosis and risk-stratified therapy are essential to mitigate CAA complications. When revascularization and thrombolysis are not feasible or unsuccessful, individualized combinations of double antiplatelet and anticoagulant therapy may reduce thrombotic risk, necessitating close multidisciplinary follow-up.

Learning points

- Incomplete Kawasaki disease can present with multivessel giant coronary aneurysms and thrombosis if diagnosis is delayed.
- Thrombolytic therapy with alteplase may fail to resolve organized thrombus in Kawasaki disease–related coronary aneurysms.
- Percutaneous coronary intervention in infants with thrombosed, aneurysmal coronaries is technically limited and often unsuccessful.

## Introduction

Kawasaki disease (KD) is the leading cause of acquired heart disease in children in developed countries.^1^ Untreated or treatment-resistant KD can lead to coronary artery aneurysms (CAAs), with the risk of thrombosis, stenosis, and myocardial ischaemia.^2^ Giant CAAs (Z score ≥10 or internal diameter ≥8 mm) carry the highest risk of adverse outcomes.^2^ Although percutaneous coronary intervention (PCI) has been attempted in select paediatric patients with KD, outcomes are variable and durability is uncertain, particularly in infants with thrombosed, fragile vessels.^3,4^ We report an infant with incomplete KD who developed multivessel giant CAAs and refractory thrombosis, resistant to alteplase thrombolysis, in whom an attempted LCX recanalization was unsuccessful. We discuss management decisions considering recent guidance and literature.

## Summary figure

**Figure:**
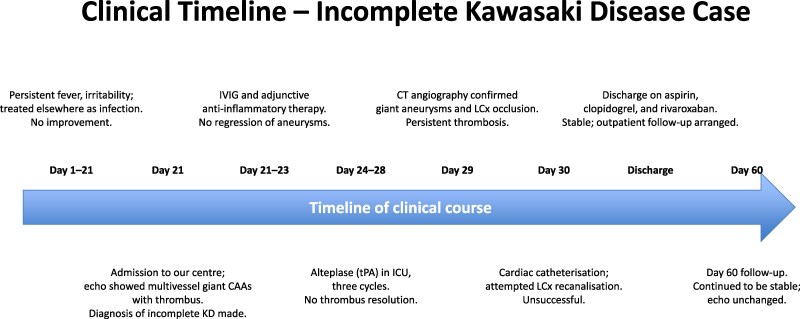


## Case presentation

**Figure 1:**
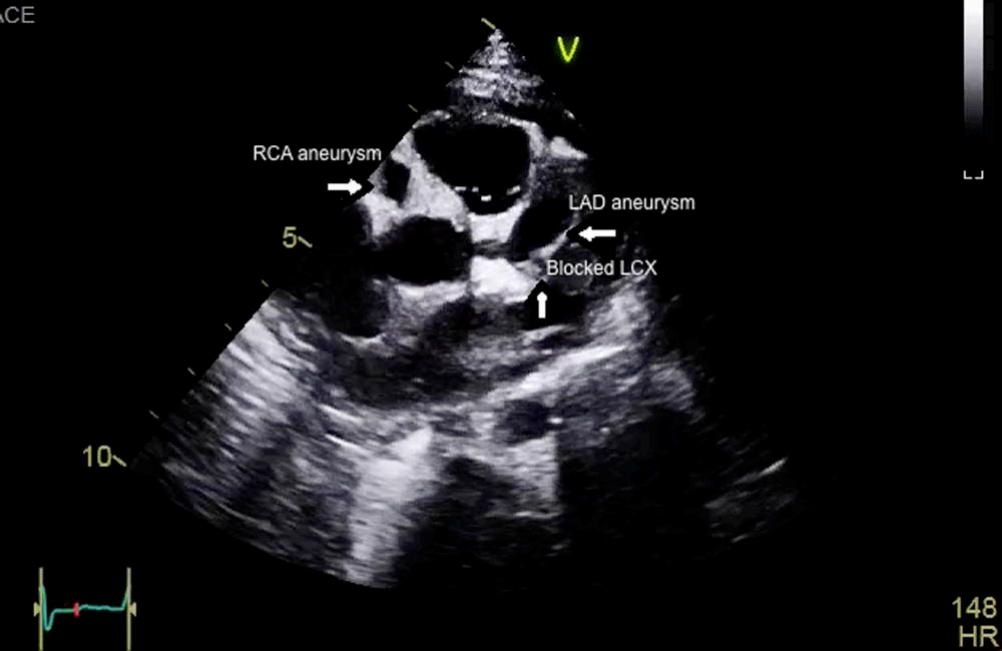
Echocardiography, short-axis view, showing aneurysms in the left anterior descending and right coronary artery, with totally thrombosed left circumflex. Arrows indicate the aneurysmal segments and the occlusive thrombus in the left circumflex artery.

**Figure 2:**
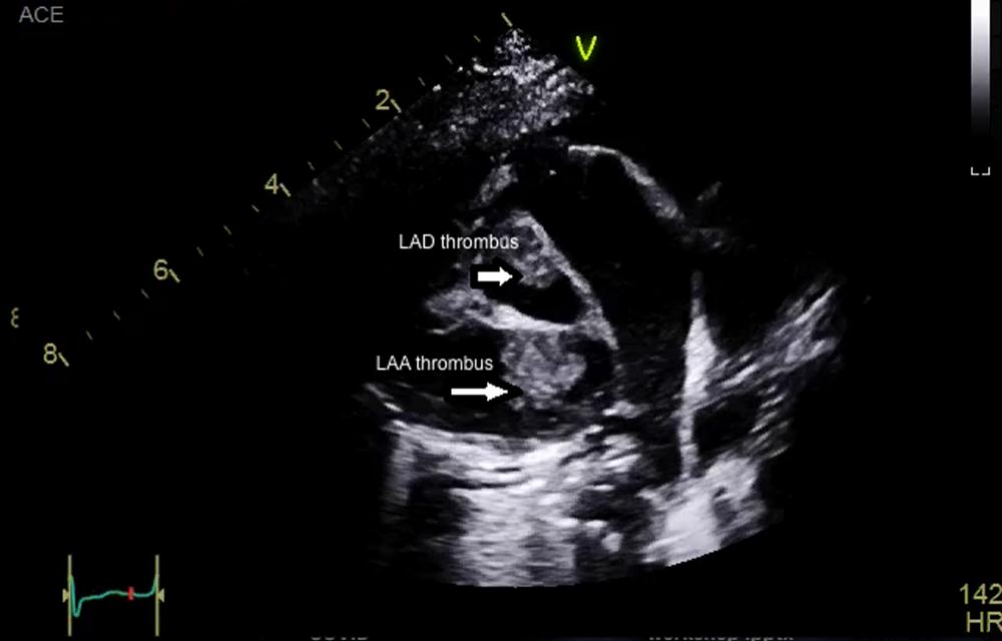
Echocardiography, long-axis view with anterior tilt, demonstrating thrombus at the left anterior descending and left atrial appendage. Arrows highlight the mural thrombus within the left anterior descending artery and the left atrial appendage.

**Figure 3:**
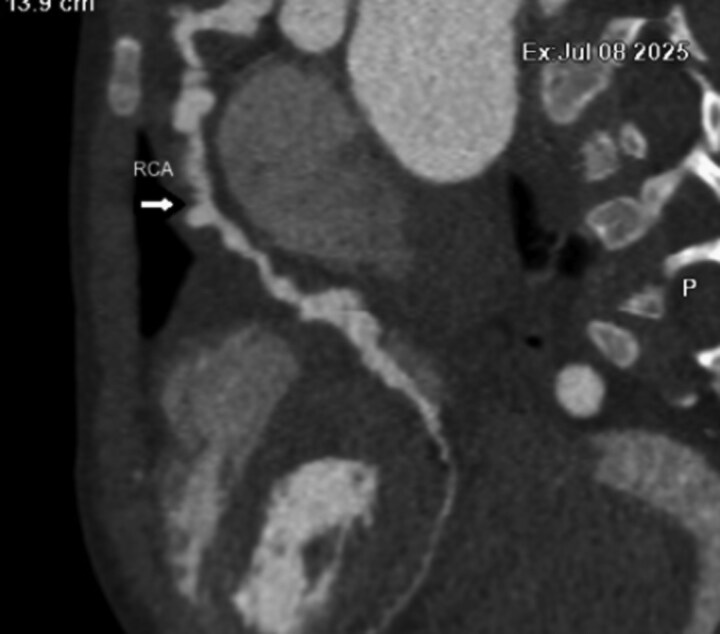
Computed tomography coronary angiography showing multiple aneurysms in the right coronary artery (RCA). The arrow indicates the RCA aneurysms.

**Figure 4:**
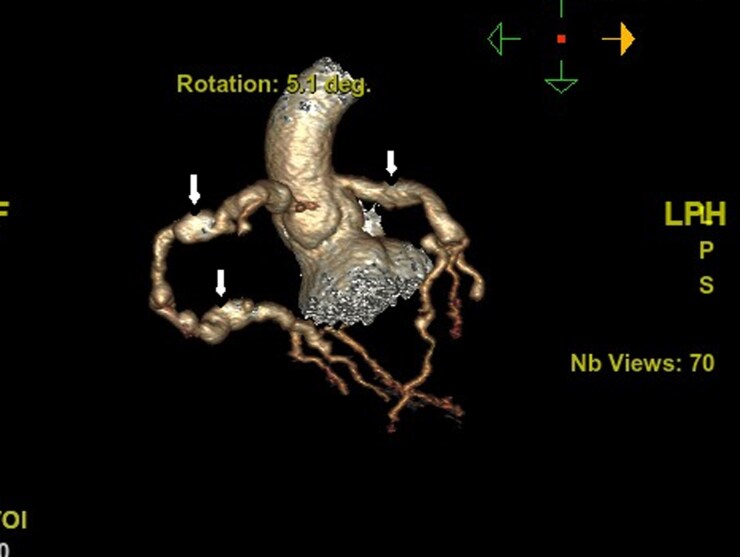
Reconstructed computed tomography image showing aneurysms involving both left anterior descending and right coronary artery. Arrows outline the fusiform and saccular aneurysms.

A 9-month-old male infant, previously well, presented with a 3-week history of persistent high-grade fever and irritability without a clear source. Initial evaluation at another hospital included antibiotics for presumed infection. On presentation to our centre, vital signs showed tachycardia; systemic examination revealed no pathognomonic mucocutaneous features of classic KD. Inflammatory markers were markedly elevated. Transthoracic echocardiography demonstrated aneurysmal dilatation of the LAD, RCA, and left circumflex (LCX) with intraluminal thrombus (*Figure 1*). On a modified long-axis view with anterior tilt, thrombus was also visualized at the LAD and left atrial appendage (*Figure 2*). CT coronary angiography confirmed giant fusiform and saccular aneurysms in all three coronaries; the RCA showed multiple aneurysms (*Figure 3*). A reconstructed CT image highlighted aneurysmal changes involving both the LAD and RCA (*Figure 4*). In the intensive care unit, alteplase thrombolysis was attempted in three cycles without clinical or imaging evidence of thrombus resolution. Treatment also included intravenous immunoglobulin and adjunctive anti-inflammatory therapy. Given ongoing concern for LCX thrombosis and ischaemic risk, cardiac catheterization was performed; wire passage and recanalization of the LCX could not be achieved. The patient remained haemodynamically stable. However, he had an initial ST-segment elevation in his ECG, with some regional wall abnormalities. He was discharged on triple antithrombotic therapy consisting of aspirin, clopidogrel, and rivaroxaban, with close outpatient follow-up.

Laboratory evaluation showed markedly elevated C-reactive protein and erythrocyte sedimentation rate, leucocytosis with neutrophil predominance, normocytic anaemia, fulfilling the American Heart Association supplemental laboratory criteria for incomplete Kawasaki disease and helping to distinguish it from other infectious or inflammatory conditions. Serial echocardiograms and a CT coronary angiography documented an occlusive thrombus filling the left circumflex coronary artery aneurysm with absent distal flow, and predominantly mural thrombi within the left anterior descending and right coronary artery (RCA) aneurysms with preserved but sluggish distal perfusion. Alteplase thrombolysis was given according to our institutional protocol for coronary thrombosis at a dose of 0.5 mg/kg/h for 6 h, repeated for three cycles, with interval echocardiographic reassessment and close monitoring of coagulation parameters, fibrinogen levels, and bleeding but without a meaningful reduction in thrombus burden. At discharge, the patient was started on triple antithrombotic therapy consisting of aspirin, clopidogrel, and rivaroxaban as an oral suspension at a body-weight–adjusted dose of 2.4 mg TDS, adapted from the scientific statement of the American Heart Association 2024.^1^ At 2-month follow-up, he remained clinically stable without ischaemic symptoms, and repeat echocardiography demonstrated persistent giant aneurysms with stable maximal diameters and thrombus burden and no new coronary or atrial thromboses.

## Discussion

This case underscores four key issues. First, delayed recognition of incomplete KD—here, a 3-week febrile prodrome before diagnosis—is associated with a higher risk of CAA formation and thrombosis.^1,2^ Contemporary guidance emphasizes early identification of high-risk patients and timely immunomodulation to prevent CAAs.^1^ Second, thrombolytic therapy with alteplase has been reported in KD patients with coronary thrombosis but results are inconsistent. Our patient showed no response despite three cycles of alteplase in the ICU, consistent with other resistant KD reports.^5,6^ Third, the role of PCI in paediatric KD remains selective. While rotational atherectomy and balloon angioplasty can relieve localized calcified stenoses in older children, restenosis and late events are not uncommon.^3,4^ In infants with acute or organizing thrombus and fragile aneurysmal segments, procedural success is limited and durability uncertain; our unsuccessful LCX recanalization reflects these constraints.^3^ Fourth, antithrombotic management for giant CAAs is evolving. Traditional regimens combine antiplatelet therapy with warfarin or low-molecular-weight heparin.^2^ Emerging evidence and recent guidance suggest that direct oral anticoagulants (DOACs) may be considered as alternatives in selected paediatric patients with giant CAAs.^7^ Given the occluding LCX and multivessel giant CAAs, our multidisciplinary team opted for triple therapy (aspirin, clopidogrel, rivaroxaban) with careful monitoring for bleeding and thrombotic complications.^7^ In our patient, warfarin was considered less attractive because of the need for frequent international normalized ratio monitoring, dietary and drug interactions, and anticipated difficulty maintaining a stable therapeutic range in early childhood, whereas long-term low-molecular-weight heparin would have required repeated subcutaneous injections and anti-factor Xa monitoring. Rivaroxaban, although off-label for Kawasaki disease-related coronary thrombosis, offered an oral, weight-adjusted regimen with emerging safety data in children with giant coronary aneurysms after Kawasaki disease and in other paediatric thrombotic indications, and was therefore selected after multidisciplinary discussion and detailed counselling of the family. Serial follow-up imaging over the first 2 months after discharge demonstrated stabilization rather than regression of aneurysm size and thrombus burden under triple therapy, underscoring the need for long-term surveillance despite apparent clinical improvement.

## Comparison with similar reports

A case report described resistant KD with giant coronary aneurysms and thrombosis, where thrombolysis and anticoagulation had limited effect, highlighting similarities with our patient.^6^ Early experiences with rotational atherectomy for calcified KD lesions achieved immediate success but showed significant late restenosis and adverse events on long-term follow-up.^3^ A recent EHJ-Case Reports article highlighted the complexity of calcified paediatric KD lesions requiring unconventional strategies.^4^ Case reports of myocardial infarction in children with KD have described emergency PCI but maintaining long-term patency is challenging.^8^ A multicentre series reported the use of DOACs in patients with giant CAAs after KD with acceptable safety,^7^ and the 2024 AHA update recognizes DOACs as alternatives to warfarin or heparin in this population.^1^ Our infant shares the feature of LCX occlusion with prior reports but differs by age, multivessel giant CAAs, failure of alteplase therapy, and adoption of DOAC-based triple therapy at discharge.^5–7^

## Conclusion

Infants with incomplete KD may develop aggressive multivessel coronary involvement if diagnosis is delayed.^1,2^ PCI may be infeasible in thrombosed, aneurysmal infant coronary arteries, and alteplase therapy may not resolve organized thrombus.^5,6^ Evolving guidance supports considering DOACs in patients with giant CAAs,^7^ though prospective paediatric data remains limited. Long-term outcomes hinge on vigilant follow-up with multimodality imaging, thrombotic risk assessment, and multidisciplinary care.^1,2^

## Lead author biography


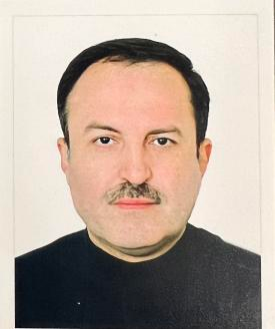


Dr. Abdelaal is a paediatric cardiologist with specialized expertise in advanced echocardiography, including 4D imaging, strain, and myocardial work in congenital heart disease. He trained as a clinical fellow at Great Ormond Street Hospital, London, and is certified by both the European Association of Cardiovascular Imaging (EACVI) and the Association for European Pediatric and Congenital Cardiology (AEPC). He currently practices at the Chest Diseases Hospital, Kuwait, where he supervise clinical care and training in paediatric echocardiography.He is interested in reporting rare cases.

**Consent:** Written informed consent for publication of this case report was obtained from the patient’s legal guardian.

## Data Availability

The data underlying this article are available within this article. No additional data are available.
